# Renin-Angiotensin System in Pathogenesis of Atherosclerosis and Treatment of CVD

**DOI:** 10.3390/ijms22136702

**Published:** 2021-06-22

**Authors:** Anastasia V. Poznyak, Dwaipayan Bharadwaj, Gauri Prasad, Andrey V. Grechko, Margarita A. Sazonova, Alexander N. Orekhov

**Affiliations:** 1 Institute for Atherosclerosis Research, Skolkovo Innovative Center, 121609 Moscow, Russia; 2 Academy of Scientific and Innovative Research, CSIR-Institute of Genomics and Integrative Biology Campus, New Delhi 110067, India; db@jnu.ac.in; 3 Systems Genomics Laboratory, School of Biotechnology, Jawaharlal Nehru University, New Delhi 110067, India; gauri080191@gmail.com; 4 Federal Research and Clinical Center of Intensive Care Medicine and Rehabilitology, 14-3 Solyanka Street, 109240 Moscow, Russia; avg-2007@yandex.ru; 5 Laboratory of Angiopathology, Institute of General Pathology and Pathophysiology, 125315 Moscow, Russia; daisy29@mail.ru; 6 Institute of Human Morphology, 3 Tsyurupa Street, 117418 Moscow, Russia

**Keywords:** atherosclerosis, CVD, RAAS, renin–angiotensin–aldosterone system

## Abstract

Atherosclerosis has complex pathogenesis, which involves at least three serious aspects: inflammation, lipid metabolism alterations, and endothelial injury. There are no effective treatment options, as well as preventive measures for atherosclerosis. However, this disease has various severe complications, the most severe of which is cardiovascular disease (CVD). It is important to note, that CVD is among the leading causes of death worldwide. The renin–angiotensin–aldosterone system (RAAS) is an important part of inflammatory response regulation. This system contributes to the recruitment of inflammatory cells to the injured site and stimulates the production of various cytokines, such as IL-6, TNF-a, and COX-2. There is also an association between RAAS and oxidative stress, which is also an important player in atherogenesis. Angiotensin-II induces plaque formation at early stages, and this is one of the most crucial impacts on atherogenesis from the RAAS. Importantly, while stimulating the production of ROS, Angiotensin-II at the same time decreases the generation of NO. The endothelium is known as a major contributor to vascular function. Oxidative stress is the main trigger of endothelial dysfunction, and, once again, links RAAS to the pathogenesis of atherosclerosis. All these implications of RAAS in atherogenesis lead to an explicable conclusion that elements of RAAS can be promising targets for atherosclerosis treatment. In this review, we also summarize the data on treatment approaches involving cytokine targeting in CVD, which can contribute to a better understanding of atherogenesis and even its prevention.

## 1. Introduction

Atherosclerosis is a serious health problem due to its relation to cardiovascular disease, which is one of the leading causes of death around the globe. Atherogenesis is based on three cornerstones: lipid metabolism alteration, inflammation, and endothelial injury.

Endothelial injury acts as an initial trigger causing the accumulation and infiltration of the multiple-modified low-density lipoprotein particles in the subendothelial space [1].

Modified LDL stimulates the production of vascular cell adhesion molecules, such as VCAM-1, P and E- selectins on the endothelial cells, which results in the recruitment of leukocytes into subendothelial space. Then, inflammatory cells migrate into the intima [2]. This process is mediated by the chemoattractant proteins, among which are MCP-1(monocyte chemoattractant proteins), and others. Macrophages, after the transformation from monocytes, express SRA, CD36, LOX-1, and other scavenger receptors, which act through modified LDL internalization. Such cells with lipid contents are called foam cells. It is important to note that cells of other types, including pericyte-like cells, can also transform into foam cells. Foam cell emergence is an initial stage of atherosclerotic lesion formation. At this stage, mast cells and foam cells along with T-lymphocytes, that migrated to the intima, release various cytokines stimulating inflammation and production of reactive oxygen species (ROS) [3,4]. Growth factors released by these cells as well as ROS stimulate smooth muscle cell migration and collagen deposition leading to the development of an atheromatous plaque [5].

ROS acts as a trigger of scavenger receptors’ expression in smooth muscle cells and also induces their transformation into foam cells. Another role of ROS in atherogenesis consists of stimulation of matrix metalloproteinases (MMPs) release, which degrades the fibrous wall of the atheromatous plaque and basement membrane of the endothelial cells. This, in turn, results in physical disruption of the plaque, which can happen by superficial erosion of the endothelial cells due to damage of their basement membrane, disruption of micro-vessels in the plaque, causing micro-hemorrhage/thrombosis, or disruption of the fibrous cap exposing the pro-thrombogenic content of the plaque. Such damage can lead to a sudden expansion of lesions and can promote arterial thrombosis [6].

## 2. RAAS System: Inflammation

**Figure 1:**
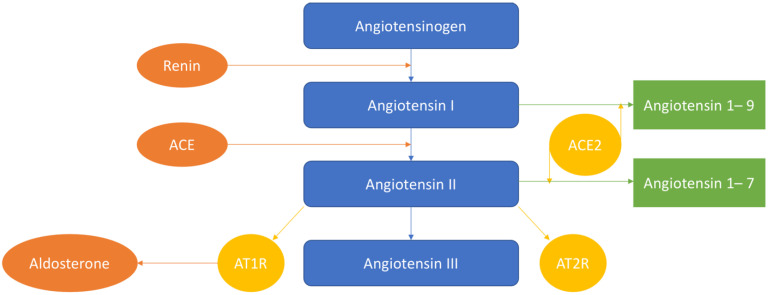
Renin cleaves angiotensinogen into 10 peptides (Angiotensin I). With the help of the angiotensin-converting enzyme (ACE), Angiotensin-I can be further cleaved to Angiotensin-II. Then, Angiotensin-I is converted to Angiotensin-1–9, and Angiotensin-II—to Angiotensin-1–7 by ACE2. Then, Angiotensin-II is converted into Angiotensin-III. Angiotensin-II acts on Angiotensin II receptor 1 (AT1R) and AT2R.

The scheme of the renin–angiotensin–aldosterone system is provided in Figure 1.

The renin–angiotensin–aldosterone system (RAAS) is a key player in inflammatory response regulation. This system manages the recruitment of inflammatory elements to the site of injury. Moreover, these inflammatory cells can, in turn, generate Angiotensin II, contributing to a local RAAS stimulation, which maintains the inflammatory cycle [7]. Angiotensin II has numerous functions, acting as a local biologically active mediator with direct effects on vascular smooth muscle cells (VSMCs) and endothelial cells, as well as a hormonal role with the renal and hemodynamic effects [8]. Moreover, Angiotensin II is a known regulator of various molecules, that are important for inflammation development. Among them, there are cytokines, chemokines, growth factors, and adhesion molecules. Thus, Angiotensin II can promote atherosclerotic plaque development through upregulating VCAM-1, ICAM-1, and P-selectin in endothelial cells and, as the consequence, in circulation [9]. This contributes to the inflammatory cell adhesion to the endothelium.

One more important molecule for the atherosclerosis development, MCP-1, may also be regulated by Angiotensin II, which can enhance its expression. MCP-1 contributes to the T-cells and monocyte recruitment to the injured site on the vascular wall. Human monocytes were showed to express less amount of MCP-1 under exposure to Angiotensin II receptor blockers [10]. Other inflammatory molecules, such as IL-6, TNF-a, and COX-2 can also be upregulated by Angiotensin II. Notably, higher CRP levels enhance the expression of the AT1 receptor in VSMC [11].

Apart from the direct effects, Angiotensin II can also affect the expression of various transcription factors, which participate in atherogenesis. Thus, Angiotensin II upregulates the expression of Nf-kB, which, in turn, regulates the expression of various genes, including cytokines, chemokines, adhesion molecules, COX-2, angiotensinogen (in a sort of feedback loop), and NOS [12].

It is undoubtful that RAAS plays an important role in the regulation of atherogenesis. This understanding gave rise to a variety of investigations highlighting the not only beneficial impact of ARBs and ACE inhibitors on blood pressure, but also their effects on the cardiovascular system due to their anti-inflammatory activities [13]. Fliser et al. have found that the serum levels of CRP, TNF-a, IL-6, and MCP-, and other markers of inflammation, along with the vascular inflammation rate in hypertensive patients was decreased during the European Trial on Olmesartan and Pravastatin in Inflammation and Atherosclerosis [14]. In studies involving animal models, it was shown that Olmesartan and losartan decreased fatty streak formation in the aorta, and, moreover, reduced the levels of the inflammatory biomarkers in atherosclerotic monkeys [15]. It is important to note that no differences were observed in blood pressure between controls and treated patients. This allows the suggestion that ARBs also have antiatherogenic effects, which are not linked to blood pressure, as we highlighted above.

Many of the atherogenic effects of Angiotensin II, such as endothelial dysfunction, cellular proliferation, and inflammation itself are mediated by impaired NO synthesis and ROS production [16].

The mechanism by which another RAAS element, aldosterone, acts during the atherogenesis, is not clear. However, the importance of aldosterone is undoubtful. This is supported by animal studies, in which aldosterone infusions into ApoE-KO mice resulted in increased size of plaques with increased plaque lipid and inflammatory cell content, similar to the vulnerable plaque in humans [17]. Although, treatment with mineralocorticoid receptor antagonists reduced the plaque development in this model [18].

## 3. RAAS System: Oxidative Stress and ROS

Oxidative stress, and especially ROS is crucially involved in the pathogenesis of atherosclerosis [5]. NADPH oxidase is, probably, one of the most significant contributors to ROS generation, which role in atherogenesis was clearly observed in murine models [19]. ROS affect endothelial phenotype and activate AKT, JAK/STAT, map kinases, and other pathways leading to the activation and proliferation of endothelial cells, and apoptosis [20,21].

The tight link between the RAAS and ROS generation has been demonstrated. Angiotensin II stimulates plaque formation at the early stages, which is one of the most crucial impacts on atherogenesis [22]. It is important to mention the ability of Angiotensin II to stimulate ROS production and at the same time to decrease the generation of NO. The mechanism through which the production of superoxide anion can be enhanced is the activation of the NADH/NADPH oxidase pathway [23]. Superoxide anion acts as a signaling molecule activating transcription of NF-kB. Subsequently, oxidative stress can modulate gene expression. Expression of MCP-1 can be upregulated, as well as leukocyte adhesion molecules (VCAM and ICAM).

The aforementioned NF-kB triggers several proinflammatory genes expression [24]. Leukocyte adhesion to the vessel wall and their infiltration to the subintimal layer are induced by proteins, whose expression is mediated by NF-kB [25]. This strictly links NF-kB to atherosclerosis initiation.

RAAS also acts through cytokine production stimulation, increasing the level of TNF-a and IL-6, which contribute to oxidative stress. These cytokines were shown to increase both mitochondrial ROS and NAD(P)H oxidase-generated ROS [26].

## 4. RAAS and Endothelial Dysfunction

The endothelium is believed to be the main element maintaining vascular function [27]. The main activities of endothelial cells are to keep a balance between vasodilatation and vasoconstriction, and also to maintain coagulation. Moreover, the endothelium is a physical barrier between the vessel itself and circulating blood. Leukocyte migration, cell proliferation, apoptosis, and other important cellular processes are regulated via various molecular mediators. The interplay between ECs and VSMCs causes the cytokines and growth factors release.

Endothelial dysfunction is suggested to be a cornerstone of RAAS implication in the pathogenesis of cardiovascular disorders. Oxidative stress is the main trigger, leading to vascular inflammation as well as endothelial dysfunction [28]. This, in turn, results in upregulation of Angiotensin II and ACE, and other parts of RAAS. There is a vicious circle affecting NO production and Angiotensin II generation. Angiotensin II acts as a direct inducer of endothelial dysfunction, which also triggers the alterations of smooth muscles previously initiated by classical cardiovascular risk factors [7]. All this results in the enhanced production of ROS lowered production of NO, and activation of other mediators of vascular disease.

Redox state, which is the crucial regulator of endothelial physiological function, can be modified by NO and other vasodilating mediators [29]. Thus, the crucial role is played by the balance between vasodilating agents and vasoconstricting mediators, or between NO and ROS, the alteration of which causes the endothelial dysfunction. Enhanced oxidative stress is associated with vasospasm, thrombosis, smooth muscle cell proliferation, and proinflammatory and pro-oxidant states [30].

Notably, NO has numerous antiatherogenic properties, not only vasodilatation action. NO may also suppress the growth and migration of VSMCs, downregulate the expression of inflammatory molecules, as well as inhibit the NF-kB activity [31,32,33].

Angiotensin II is a known regulator of redox state, through which it affects endothelium. An example of such action is an expression of the VSMC proinflammatory phenotype, which stimulates significant proinflammatory background by activating the transcription factor NF-kB, stimulating the expression of VCAM and the release of the IL-6 and TNF-α [34,35]. Such pro-inflammatory impact on vessel wall cells and endothelial cells interacts with the various risk factors, including diabetes and dyslipidemia, leading to increased inflammation, and thus leading to atherosclerosis development.

## 5. Other Actions of RAAS

Angiotensin II plays an important role in vascular remodeling. It is able to stimulate plaque formation by regulating the extracellular matrix composition, survival of VSMCs, and cell migration [8]. The release of metalloproteinases and other components of the extracellular matrix may be induced by Angiotensin II. In addition, Angiotensin II plays a role of growth factor itself and stimulates the growth factors expression, such as insulin-like growth factor, platelet-derived growth factor, and others in a local autocrine pathway [36]. Thus, RAAS acts as a central element of vascular remodeling and lesion formation via local actions.

Modulation of the coagulative state is another crucial action that is modulated by Angiotensin II. The formation of PAI-1 is triggered by Angiotensin II via exposure to specific angiotensin endothelial receptors [37]. Moreover, ACE has an ability to downregulate the production of tissue plasminogen activator (tPA), acting through bradykinin degradation. Bradykinin is a powerful trigger of tPA synthesis in endothelial cells, which leads to thrombus formation via the induction of a procoagulative state [38].

Actual studies reveal the existence of a positive feedback mechanism for local angiotensin formation [39]. RAAS molecules can be produced by inflammatory cells. Chymase can be secreted by mast cells, cathepsin G can be produced by neutrophils, and Angiotensin II and ACE—by macrophages [40]. Notably, oxidized LDL-induced macrophage activation has the potential to enhance the expression of ACE, which, in turn, increases activation of RAAS [41]. Angiotensin II receptors on cells of various types are activated within by increased local levels of tissue ACE and Angiotensin II the atherosclerotic lesion. The result of this activation is a progression of the lesion via the foam cell formation along with VSMCs proliferation. This makes plaques more vulnerable to rupture and thrombosis [42].

An increased expression of Angiotensin II and ACE contributes to the relation of RAAS activation to atherosclerosis progression [43]. The study on human carotid artery endarterectomy specimens revealed the association between the RAAS activity enhancement and vulnerable plaques formation [44]. Notably, no signs of very increased levels of local RAAS components in inflamed plaque prone to rupture were found. Thus, enhanced ACE and Angiotensin II generation can play an important role in the development of acute ischemic vascular diseases via their actions on extracellular matrix composition and metalloproteinases production. Metalloproteinases may contribute to the vulnerability of atherosclerotic lesions, especially in the area at increased circumferential and shear stress, and enhancing the probability of local rupture.

## 6. Targeting Potential

Atherosclerosis is a complex disease, which implies numerous molecular components. All known components have different potential as a therapeutic target, and the search for the best target remains the crucial line of research. In Table 1 we summarized the data about targeting the RAAS system in the treatment of various cardiovascular conditions, which can be important for further atherosclerosis-related investigations.

### 6.1. RAAS Inhibition in Clinical Trials

Results of a number of randomized case-control trials have shown that inhibitors of RAAS may have beneficial effects on patients suffering from atherosclerosis not only because of their anti-hypertensive properties but also through other effects (pleiotropic effects). It was observed, that the blood pressure lowering is undoubtfully beneficial for hypertensive patients, but it does not matter which drug is used to achieve these results. RAAS inhibitors were compared to classical antihypertensive drugs (such as Ca2+-channel blockers) and no difference in their effects was shown. The STOP2 (the Swedish Trial in Old Patients with Hypertension 2) trial demonstrated no difference between anti-hypertensive drugs. The large RCT ALLHAT (the Antihypertensive and Lipid-Lowering Treatment to Prevent Heart Attack Trial) trial revealed no decrease of myocardial infarctions but neither no enhance of stroke in patients who received lisinopril in comparison with those who received amlodipine. Although, the LIFE (the Losartan Intervention for Endpoint Reduction in Hypertension Study) trial demonstrated a marked decrease of stroke risk, but not of myocardial infarction in hypertensive patients with left ventricular hypertrophy who received losartan in comparison to a patient who received atenolol.

The results of EUROPE (efficacy of perindopril in reduction of cardiovascular events among patients with stable coronary artery disease) and the HOPE (Heart Out- comes Prevention Evaluation) trials contribute to the decreased risk of myocardial infarction with ACE inhibitors. Moreover, such a result was greater than expected from blood pressure reduction. Due to the fact that patients in the HOPE and EUROPA trials suspectedly had more advanced atherosclerosis, it is possible that the role in improving the prognosis of ACE inhibitor might be less important in patients at lower cardiovascular risk and without overt coronary heart disease [20].

### 6.2. Angiotensin-1–7

Angiotensin-1–7 was shown to stimulate anti-inflammatory phenotypes, contributing to the suppression of vascular lipid accumulation [54]. Investigation on ApoE-KO mice has shown that treatment with Ang-1–7 can decrease the macrophage infiltration, oxidative stress because of the reduction in Nox4, which is an important subunit of the NADPH oxidase complex [45]. Results of another investigation demonstrated that the expression of TNF-α, IL-6, and other pro-inflammatory cytokines was significantly reduced in response to Ang-1–7 administration in both aortic plaque and macrophages from ApoE-KO [55]. Moreover, the pretreatment with a MasR agonist AVE0991 appeared to decrease the level of IL-2 and activated CD4+ T cells [56,57]. Taken together, these data contribute to the atheroprotective effect of the Ang-1–7/MasR pathway.

Ang-1–7 was also observed to suppress the VSMCs migration and proliferation unlike the Ang-II-induced proliferative and hypertrophic effects [58]. Ang-1–7 was also a subject of investigation of Yang and colleagues, who found out the inductive effect of Ang-1–7 on MasR/ERK1,2/p38 and MasR/JAK/STAT pathways in VSMCs, which contributed to plaque formation. Moreover, Ang-1–7 has shown a potential to negatively regulate vascular fibrosis, as can be noticed by decreasing in matrix metalloproteases (MMP) MMP-2/MMP-9 in atherosclerotic plaques [45].

Therefore, Ang-1–7 treatment indicated (1) a reduction in the neointimal layer growth due to endothelium structural recovery (2) and an atheroprotective properties appearance caused by AT2R and MasR binding [59]. Additionally, Ang-1–7 reduces atherosclerotic lesion formation as activation of AT2R provided low collagen accumulation. However, it was also revealed that administration of Ang-1–7 leads to the accumulation of collagen and increases plaque stability [45]. Analogically, the A77 treatment, which is an Ang-1–7 antagonist, promotes low plaque stability and low collagen levels [54].

Besides, the heptapeptide can act like a β-arrestin-biased AT1R agonist without causing the activation of the Gq subunit due to the additional antihypertensive effect related to this peptide [46,59].

It is noteworthy that the plasmatic Ang-1–7 increase influences the regulation of lipid metabolism, reducing triglycerides, cholesterol, adipose tissue, and improving glucose metabolism [47]. The scientists consider the influence of adiponectin in the regulation of the glucose and lipid metabolism induced by Ang-1–7. As a matter of curiosity, the rejection of MacR achieved the opposite effect: cholesterol and triglyceride levels increased, and carbohydrates negatively affected the metabolism [47].

### 6.3. ACE-2

ACE2 has already attracted attention as a potential target for atherosclerosis treatment, and therapeutic strategies involved both newly discovered drugs and compounds known in clinical practice. For example, overexpression of ACE2 in THP-1 cells and primary monocytes caused by plasmid-mediated transfection was shown to result in significantly lowered ACE2 mRNA expression. What is more, gene expression of cellular adhesion molecules, such as MCSF (macrophage colony-stimulating factor), VCAM-1, and ICAM-1, was enhanced, and pro-atherogenic phenotype was triggered. Losartan and captopril demonstrated an ability to reduce, at least in part, these effects [60]. In high-cholesterol fed rabbits losartan was observed to suppress the progression of atherosclerotic plaques and stimulate the expression of ACE2 within the plaques [61]. Moreover, losartan appeared to inhibit Ang-II-induced reduction of both ACE2 protein and activity in smooth muscle cells in vitro. Taken together, these data suggest that ACE inhibitors or AT1R antagonists can stimulate ACE2 expression and beneficially affect its atheroprotective properties.

Apart from antagonists of ACE2 blockers, there are also ACE2-activating compounds, including DIZE (diminazene aceturate) [48]. DIZE has beneficial effects on lipid metabolism and the cardiovascular system in various models [49,50,62]. Notably, DIZE was demonstrated to increase the stability of atherosclerotic plaques in ApoE-KO mice and lower the VCAM-1 and ICAM-1 expression [51,63].

### 6.4. Angiotensin-II

Ang-II is the main effector of the renin–angiotensin–aldosterone system [64]. The effects of Ang-II are mediated by its binding into the Angiotensin Type 1 and Type 2 receptors (AT1R and AT2R, respectively). Inhibitors of ACE and AT1R blockers (ARBs) were shown to induce anti-atherosclerotic effects in clinical and preclinical experiments [65]. Enalapril appeared to regulate the antioxidant defense system, lower inflammatory mediators, and inhibit NADPH oxidase activity in apolipoprotein E-deficient mice [52]. Olmesartan (an ARB), was shown to markedly decrease vascular inflammation in hypertensive patients. In addition, serum levels of TNF-α, IL-6, CRP (C-reactive protein), and other markers of inflammation were observed to be reduced [20]. Long-term therapy with valsartan reversed atherosclerosis in individuals with thickening of the carotid wall, while inflammation and oxidative stress were reduced, and peripheral smooth muscle function improved [66].

Moreover, long-term therapy with valsartan has been associated with atherosclerosis regression in individuals with thickening of the carotid wall. These effects were accompanied by concomitant improvements in oxidative stress markers, inflammation, and peripheral smooth muscle function [43].

### 6.5. Aldosterone

Because of the multiple implications of aldosterone in the pathogenesis of atherosclerosis, it seems to be promising to use agents which block aldosterone action in patients with CAD. The theory of “aldosterone breakthrough” exists, which suggests that long-term ACE inhibition or ARB therapy alone does not completely inhibit the generation of aldosterone. This can be partially explained by the suppression of the normal negative feedback mechanisms and leading to the reactivation of AngII signaling. What is more, the generation of aldosterone is stimulated by not only AngII but other triggers, such as potassium, too [43].

Blockade of mineral-corticoid receptor (MR) was shown to be effective for heart failure in clinical trials. The 30% decrease of mortality risk was demonstrated in severe heart failure, with no difference between ischemic and non-ischemic cases, in the RALES study [67]. In the EPHESUS study elprenone, the blocker of MR, was used in complex with ACE inhibitors and other components of standard therapy 3 to 14 days post-myocardial infarction associated with left ventricular dysfunction and congestive heart failure. This study reported a 15% decrease in risk of all-cause mortality and a 13% decrease in risk of the composite endpoint of CV mortality/CV hospitalization [53]. Post-hoc analysis revealed an early benefit of 31% already recognizable at 30 days post-randomization. In another study, EMPHASIS-HF faced an early recruitment stop due to the meeting predefined efficacy endpoints. The primary end point composed of CV death or hospitalization for heart failure occurred in 18.3% of patients in the eplerenone group as compared with 25.9% in the placebo group, suggesting benefit in mildly symptomatic patients, too. [68]

However, the recent analysis of EPHESUS data revealed a worrying issue considering hyperkalemia, which can restrain clinicians from the use of blockers of aldosterone. This study indeed showed a 4.4%/1.6% elevation of incidence of hyperkalemia with the administration of eplerenone. However, with periodic monitoring of serum K+ and correcting eplerenone dosage as needed, this did not affect the clinical benefit on mortality [69].

Summarizing data from the aforementioned trials, the beneficial effect of targeting aldosterone in patients with heart failure seems to be proven. However, it is still unclear, if it works for CAD patients without heart failure. ALBATROSS trial revealed no beneficial effect of aldosterone blockade with spironolactone in acute myocardial infarction [70].

An alternative to the approach which is described above is targeting aldosterone itself in contrast to the targeting of aldosterone. Aldosterone synthase inhibitors are also being developed for the treatment of various conditions, such as primary hyperaldosteronism, hypertension, and heart failure. If effective, it would be beneficial to examine these agents also in acute coronary syndromes or other manifestations of atherosclerosis [71].

### 6.6. Renin

In the middle of the XX century, the crucial role of renin in RAAS controlling was evaluated. Due to this, renin was considered to be a promising target for cardiovascular protection [43]. However, all attempts to inhibit renin directly resulted in no beneficial effect which could be used in clinical practice. This can be explained by various technical issues, including low oral bioavailability and insufficient affinity for renin active site, which suppress the progress of investigations [72].

Luckily, these problems were solved with the development of aliskiren, which was the first direct inhibitor of renin that progressed to and passed phase III studies. Both the European Medicines Agency and the US Food and Drug Administration approved aliskiren for the treatment of hypertension. Several studies have proven the efficacy of aliskiren for blood pressure lowering. The aliskiren effect was shown to be greater than the effect of placebo or hydrochlorothiazide and also of ACE inhibitor or ARB. The low frequency of adverse effects was reported for the aliskiren, which allows the conclusion that this drug is well-tolerated [73].

However, atheroprotective effects of aliskiren were only shown in preclinical settings. The size of atherosclerotic plaques was shown to be reduced in mice who received aliskiren, and the plaque phenotype was also shown to be more stable [74]. Almost the same effect was observed in rabbits who received aliskiren: impaired nitric oxide bioavailability was improved and the size of plaques decreased [75].

Interesting results were obtained in the ALPINE study. MRI quantification of atheroma plaque burden demonstrated that aliskiren use in patients with preexisting cardiovascular disease resulted in an unexpected increase in aortic atherosclerosis compared with placebo. Such results may have implications for the use of renin inhibition as a therapeutic strategy in patients with cardiovascular disease, especially in those receiving ACEI/ARB therapy [76].

## 7. Conclusions

The renin–angiotensin–aldosterone system has various implications in atherogenesis at all stages. RAAS impact starts from the very beginning of lesion formation and accompanies the pathogenesis to advanced plaque development.

Elements of RAAS acts each in their own time. Angiotensin II is probably the most intriguing molecule in this system due to its importance for inflammation, ROS generation, and endothelial dysfunction. However, one of the most promising treatment targets appeared to be ACE2. Losartan, captopril, and DIZE were found to have beneficial effects on various aspects of atherosclerosis development in ApoE-KO mice and other animal models due to their ability to regulate ACE2. Angiotensin-1–7 appeared to exhibit atheroprotective properties by itself by reduction of proinflammatory molecule levels and by oxidative stress suppression.

Despite the promising findings in animal models, there are still numerous white gaps in understanding of the mechanisms by which RAAS elements act in atherosclerosis pathogenesis. This makes RAAS an important subject for further fundamental research and therapeutic strategies approaches.

## Figures and Tables

**Table 1:** Targeting various elements of the RAAS system in the therapy of cardiovascular disorders.

Drug	Target	Effect	Study	Reference
Angiotensin-1–7	RAAS	decrease the macrophage infiltration, oxidative stress	ApoE-KO mice	[37]
Losartan	ACE2	gene expression of cellular adhesion molecules enhancement	high-cholesterol fed rabbits	[45]
DIZE	ACE2	the stability of atherosclerotic plaques increase	ApoE-KO mice	[46,47]
Enalapril	Angiotensin-II	regulate the antioxidant defense system, lower inflammatory mediators, and inhibit NADPH oxidase activity	ApoE-deficient mice	[48]
Olmesartan	Angiotensin-II	decrease vascular inflammation	Hypertensive patients	[49]
valsartan	Angiotensin-II	atherosclerosis regression	Individuals with thickening of the carotid wall	[50]
Elprenone	Aldosterone (MR)	15% decrease of risk of all-cause mortality and 13% decrease of risk of the composite end point of CV mortality/CV hospitalization	Post-myocardial infarction associated with left ventricular dysfunction and congestive heart failure	[51]
spironolactone	Aldosterone (MR)	no beneficial effect	Acute myocardial infarction	[52]
aliskiren	Renin	Approved drug for blood pressure lowering	Hypertension	[53]

## Data Availability

Not applicable.
